# Analyses on the Infection Process of Rice Virus and the Spatiotemporal Expression Pattern of Host Defense Genes Based on a Determined-Part Inoculation Approach

**DOI:** 10.3390/pathogens11020144

**Published:** 2022-01-24

**Authors:** Wei Guo, Chenyang Li, Bo Zeng, Jie Li, Zhaoyun Wang, Shuhui Ma, Linlin Du, Ying Lan, Feng Sun, Chengye Lu, Shuo Li, Yijun Zhou, Yunyue Wang, Tong Zhou

**Affiliations:** 1 State Key Laboratory for Conservation and Utilization of Bio-Resources in Yunnan, Ministry of Education Key Laboratory of Agriculture Biodiversity for Plant Disease Management, College of Plant Protection, Yunnan Agricultural University, Kunming 650201, China; guoweiau@163.com (W.G.); luchengye1995@sina.com (C.L.); 2 Key Laboratory of Food Quality and Safety, Institute of Plant Protection, Jiangsu Academy of Agricultural Sciences, Nanjing 210014, China; chenyangli@zju.edu.cn (C.L.); lijie_0913@126.com (J.L.); wangzhaoyundyx@126.com (Z.W.); 2020802240@njau.edu.cn (S.M.); 20130050@jaas.ac.cn (L.D.); 20100045@jaas.ac.cn (Y.L.); sunfeng@jaas.ac.cn (F.S.); lishuo@jaas.ac.cn (S.L.); yjzhou@jaas.ac.cn (Y.Z.); 3 National Agricultural Technology Extension and Service Center, Beijing 100125, China; zengbo@agri.gov.cn; 4 Department of Plant Pathology, College of Plant Protection, Nanjing Agricultural University, Nanjing 210095, China; 5 Jiangsu Academy of Agricultural Sciences Joint Laboratory, International Rice Research Institute, Nanjing 210014, China

**Keywords:** *Rice stripe virus*, *Rice black streaked dwarf virus*, small brown planthopper, viral infection process, spatiotemporal expression of defense genes

## Abstract

Rice viral diseases adversely affect crop yield and quality. Most rice viruses are transmitted through insect vectors. However, the traditional whole-plant inoculation method cannot control the initial inoculation site in rice plants because the insect feeding sites in plants are random. To solve this problem, we established a determined-part inoculation approach in this study that restricted the insect feeding sites to specific parts of the rice plant. *Rice stripe virus* (RSV) was used as the model virus and was inoculated at the bottom of the stem using our method. Quantitative real-time PCR and Western blot analyses detected RSV only present at the bottom of the Nipponbare (NPB) stem at 1 day post-inoculation (dpi), indicating that our method successfully controlled the inoculation site. With time, RSV gradually moved from the bottom of the stem to the leaf in NPB rice plants, indicating that systemic viral spread can also be monitored using this method. In addition, a cultivar resistant to RSV, Zhendao 88 (ZD88), was inoculated using this method. We found that RSV accumulation in ZD88 was significantly lower than in NPB. Additionally, the expression level of the resistant gene *STV11* in ZD88 was highly induced at the initial invasion stage of RSV (1 dpi) at the inoculation site, whereas it remained relatively stable at non-inoculated sites. This finding indicated that *STV11* directly responded to RSV invasion to inhibit virus accumulation at the invasion site. We also proved that this approach is suitable for other rice viruses, such as *Rice black-streaked dwarf virus* (RBSDV). Interestingly, we determined that systemic infection with RSV was faster than that with RBSDV in NPB, which was consistent with findings in field trails. In summary, this approach is suitable for characterizing the viral infection process in rice plants, comparing the local viral accumulation and spread among different cultivars, analyzing the spatiotemporal expression pattern of resistance-associated genes, and monitoring the infection rate for different viruses.

## 1. Introduction

Rice, a staple food consumed globally, is crucial for ensuring worldwide food security [1,2]. Rice virus disease seriously threatens the guarantee of the yield and quality of rice every year and causes huge economic losses. *Rice stripe virus* (RSV) is the most widespread and destructive rice virus. Before 2010, more than 80% of rice fields in Eastern China and South Korea were affected by this disease every year [3,4,5,6,7,8,9]. RSV belongs to the genus *Tenuivirus* and is transmitted by the insect vector small brown planthopper (*Laodelphax striatellus*, SBPH) [6,10,11,12,13]. SBPH can also transmit *Rice black-streaked dwarf virus* (RBSDV), a member of the *Fijivirus* genus of the Reoviridae family [14]. In addition to rice, RBSDV can also infect gramineous crops, such as wheat, barley, oats, corn, and sorghum, causing serious economic loss by reducing crop productivity [4,15,16,17,18,19,20].

Viruses are obligate parasites that rely on living host cells to complete their lifecycles [21]. Therefore, establishing effective artificial inoculation methods is crucial for research on virology, host-virus interactions, and host resistance analyses. Most rice viruses are insect-borne viruses that are transmitted by insect vectors [22]. Plant viruses are generally inoculated through micro-wounds, mechanical friction, infectious clones, or viruliferous insect feeding [23,24,25,26]. Due to the lack of established infectious clones, artificial inoculation methods for rice viruses are limited, and the currently used whole-plant inoculation model uses viruliferous insects that feed on rice plants [26].

This whole-plant inoculation method has been widely used in rice-virus interaction studies and rice resistance analyses [26]. Using this method, Wang et al. showed that *STV11* (RAP-DB: *LOC_Os11g30910*) is a resistance gene for RSV in rice, which is upregulated upon RSV infection; it can sulfonate salicylic acid (SA) into sulphonated SA (SSA) to confer durable RSV resistance. A protoplast transfection assay also demonstrated that *STV11* inhibited RSV replication [5]. However, because the feeding of insects is random, it is difficult to distinguish and characterize the local accumulation and systemic infection process of rice viruses. Therefore, studies of resistance mechanisms in rice are mainly conducted at whole-plant level. Whether the resistance gene, such as *STV11*, inhibits viral replication or spread at the plant level is still unclear, and the spatiotemporal expression patterns of resistance genes or defense genes in different tissues of rice upon viral infection remain to be investigated. To address these limitations, a method to inoculate the virus on a determined-part of rice plant is required.

In this study, RSV and RBSDV transmitted by SBPH were used as models of rice viruses, and a determined-part inoculation approach was established to limit viruliferous insects to the bottom of the stem of rice plants. Transmitting of the virus in this manner controls the initial viral infection area. Molecular biology and immunological methods were used to detect the initial invasion of the viruses. The accumulation of viruses at the invasion site and the spread of viruses in plants were also characterized. In addition, the RSV-resistant cultivar Zhendao 88 (ZD88) was inoculated using this approach, and spatiotemporal expression of the resistance gene *STV11* was explored. This approach can be used to study the accumulation and spreading mechanism of viruses in rice plants and to clarify the antiviral defense mechanisms in rice plants.

## 2. Results

### 2.1. A Determined-Part Inoculation Approach to Detect the Infection Process of Rice Stripe Virus (RSV)

**Figure 1:**
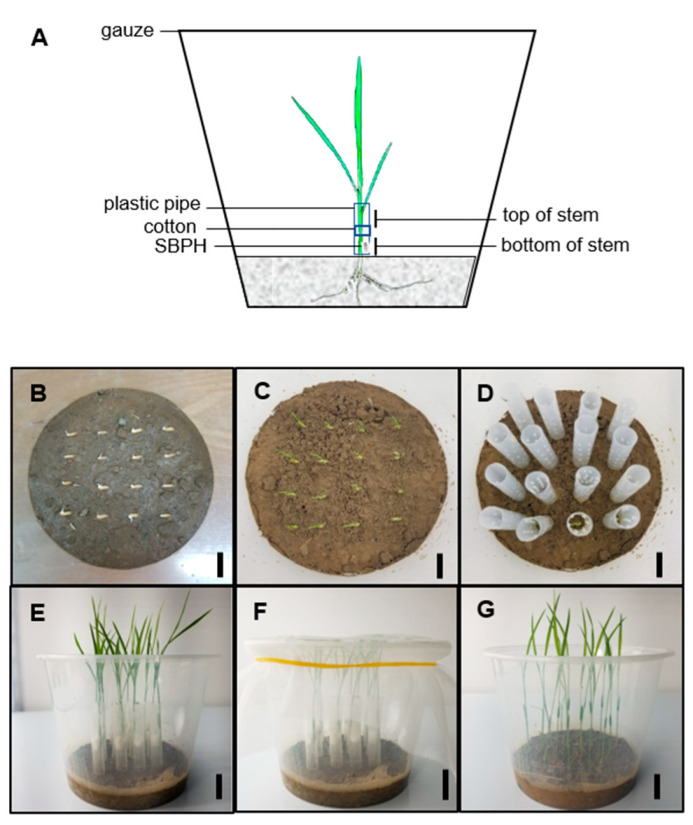
Graphic illustration of rice virus inoculation method in a specific site on rice plants. (**A**) Model of inoculating virus at the bottom (2 cm from soil surface) of the plant stem. (**B**–**G**). Photographs of the process of inoculating rice virus in a determined site. Rice seeds were placed in a Petri dish to soak seeds to accelerate germination. After germination, they were sown in 1.5 L disposable plastic pots with a round bottom of 14 cm diameter. Sixteen seeds were sown in each pot at a spacing of 2 cm (**B**). After the rice seedlings grew for 3 days (**C**), a transparent plastic tube of 1.5 cm diameter and 5 cm length with holes on the tube wall was placed over it (**D**) to make the rice seedlings grow along the tube. When the seedlings grew to 1.5 leaf age (10 d after sowing), the viruliferous rate of SBPH was tested, the actual number of SBPH required was calculated according to the effective inoculation number per plant, and the SBPH was placed at 2.0 cm base of the rice seedling stem. Cotton was used to block the position above 2.0 cm of the stem base (**E**) and the pot was covered with gauze after inoculation (**F**). After 3 days of inoculation, all SBPHs were removed, and the plastic tubes were removed. The rice seedlings were placed in a greenhouse at 28–30 °C and grown under artificial light with a 14 h light/10 h dark cycle (**G**). Scale bars, 2 cm for (**B**–**G**).

**Figure 2:**
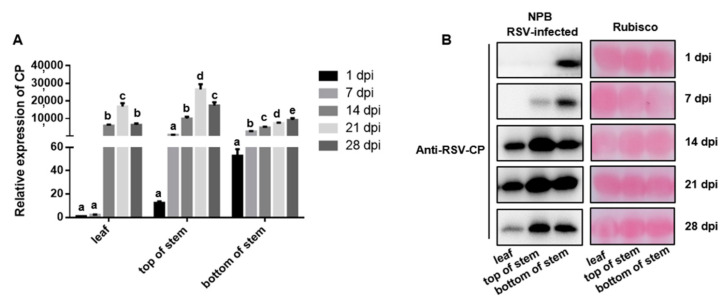
*Rice stripe virus* (RSV)-CP accumulation in different parts of Nipponbare (NPB). RSV was inoculated on the bottom stem of NPB by the determined-part inoculation method, and the transcription and accumulation levels of RSV-CP were detected during infection. (**A**) Relative expression of RSV-CP in different parts of NPB plants based on quantitative real-time polymerase chain reaction (qRT-PCR) analyses in which the average expression level of RSV-CP in the leaves at 1 dpi was set as 1 to estimate relative levels of gene expression in other parts of plants. (**B**) The expression level of RSV-CP in different parts of NPB plants by Western blot. All data are shown as mean values ± SD error bars; different letters on bar graphs indicate significant differences by Duncan’s multiple range tests; *p* < 0.01.

To verify whether the determined-part inoculation approach can control the initial invasion of the rice virus, the Nipponbare (NPB) was inoculated with RSV by using the determined-part inoculation method at the bottom of the stem (Figure 1) (SBPHs usually like to eat the stem in the field [27]) or by using the traditional whole-plant inoculation method [3,26]. The different parts of the rice plants were then collected for Western blot analysis. The results showed that the accumulation of RSV-CP was only detected at the inoculation position 1 day after RSV inoculation at the bottom of the NPB stem (Figure 2B), whereas in plants inoculated by a whole-plant inoculation method, the accumulation of RSV-CP was detected in all parts of the plant (Supplemental Figure S1). This suggests that SBPHs could transmit the virus to the stem and leaf of the plant. In addition, the determined-part inoculation approach can accurately regulate the initial invasion site of the rice virus and lead to successful invasion.

RSV was inoculated on the bottom stem of NPB using the determined-part inoculation approach, and the transcription and accumulation levels of RSV-CP were detected during the infection process. The results showed that from the initial day of inoculation, the accumulation of RSV-CP at the bottom of the stem of NPB plants increased significantly from the previous time point every 7 days thereafter, and reached a peak at 28 days after inoculation (Figure 2A). RSV-CP at NPB-inoculated sites showed a continuous growth trend, indicating that this approach can demonstrate the accumulation of RSV-CP at the invasion site.

RSV-CP protein accumulation was detected at the bottom of the NPB stem on day 1 after inoculation, and at the top of the NPB stem and leaf on days 7 and 14 after inoculation, respectively (Figure 2B). The transcription level showed the same trend (Figure 2A). With an increase in time, RSV-CP gradually moved from the bottom of the stem to the leaf in NPB rice plants (Figure 2B), which indicated that RSV had spread in the plant, and this approach can show the spread of the virus from the invasion site to other parts of the plant.

In conclusion, inoculation of rice plants with RSV using this approach can accurately control the initial infection area of the virus. After the virus successfully infects the rice plant, the infection process of the virus in different parts of the plant can be detected.

### 2.2. Comparison of Virus Accumulation and Spread between Resistant and Susceptible Cultivars

**Figure 3:**
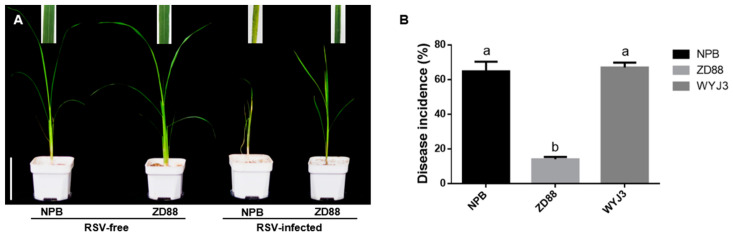
Resistance of the tested cultivars to RSV are indicated by symptoms and disease incidence. (**A**) *Rice stripe virus* (RSV) symptoms on NPB and Zhendao88 plants. Scale bar, 10 cm. (**B**) Disease incidence in NPB and Zhendao88 plants following RSV inoculation after 28 days. Wuyujing No. 3 (WYJ3) was used as the susceptible control. All data are shown as mean values ± SD error bars. Different letters on bar graphs indicate significant differences by Duncan’s multiple range tests; *p* < 0.01.

**Figure 4:**
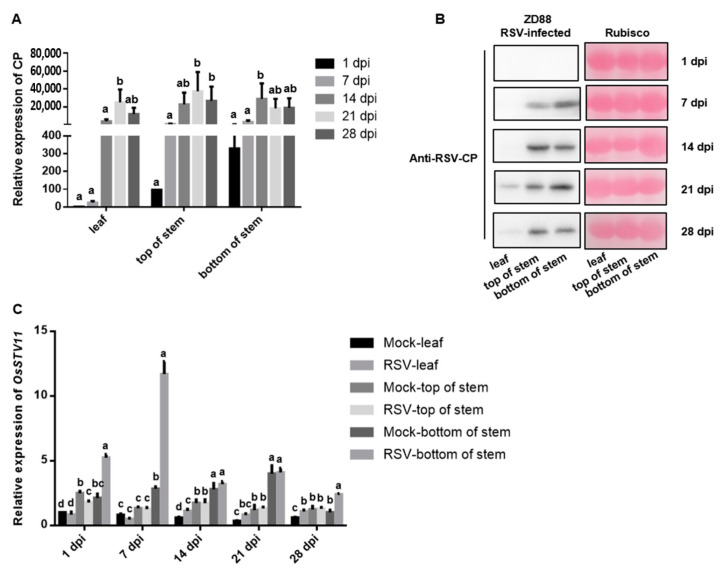
RSV-CP accumulation and spatiotemporal expression of RSV resistance genes *OsSTV11* in different parts of Zhendao88 after RSV infection. (**A**) Relative expression of RSV-CP in different parts of ZD88 plants based on qRT-PCR analyses in which the average expression level of RSV-CP in the leaves at 1 dpi was set as 1 to estimate relative levels of gene expression in other parts of plants. (**B**) The expression level of RSV-CP in different parts of ZD88 plants by Western blot. (**C**) Relative expression of *OsSTV11* in different parts of RSV-free and RSV-infected ZD88 plants based on qRT-PCR analyses in which the average expression level of *OsSTV11* in the leaves of mock plants at 1 dpi was set as 1 to estimate relative levels of gene expression in other parts of plants. All data are shown as mean values ± SD error bars. Different letters on bar graphs indicate significant differences by Duncan’s multiple range tests; *p* < 0.01.

Zhendao 88 (ZD88) and NPB were used as RSV-resistant and RSV-susceptible cultivars, respectively, to compare the differences in virus accumulation between these two cultivars. NPB plants infected with RSV showed yellow-green leaf color and new leaf curl at the 28th day post-inoculation (dpi), which was more serious than those symptoms in ZD88 (Figure 3A), and the disease incidence rate of NPB plants was also significantly higher than that of ZD88 (Figure 3B). Similarly, RSV was inoculated on the bottom stem of ZD88 using this approach, and the results showed that no coat protein accumulation was detected in ZD88 plants, whereas RSV-CP was detected in NPB-inoculated sites 1 day after inoculation (Figure 2 and Figure 4A,B). On the 7th day after inoculation, RSV-CP transcription and protein accumulation were detected at the top of the stems of these two cultivars, indicating that RSV can successfully invade NPB and ZD88 (Figure 2 and Figure 4A,B). The transcription and protein accumulation of RSV-CP were detected in the leaves of NPB and ZD88 on days 14 and 21 after inoculation, and the difference reached a significant level (Figure 2A and Figure 4A). RSV spread in both resistant and susceptible cultivars. The difference was that RSV-CP accumulation in ZD88 was significantly lower than that in NPB (Figure 2B and Figure 4B), indicating that virus accumulation in ZD88 was suppressed.

Moreover, the RSV-CP transcription levels in each part of ZD88 and NPB were compared at different time points. The results showed that except for the significant difference in viral transcripts at the inoculation site of NPB and ZD88 on the first day post-inoculation, at other time points, the viral transcription levels in NPB were significantly higher than those in ZD88 in all parts (*p* < 0.05) (Supplemental Figure S2), which indicated that the accumulation of the virus in ZD88 was inhibited.

### 2.3. Spatiotemporal Expression Analysis of STV11 in ZD88

Previous studies have found that *STV11* is an RSV resistance gene that inhibits the replication of RSV, and is induced upon RSV infection. However, the infection stage of *STV11* is induced, and whether the expression of *STV11* responds to RSV infection in all parts of the rice plant remains to be investigated. To analyze the spatiotemporal expression pattern of *STV11* upon RSV infection, the determined-part inoculation approach was used for ZD88 with RSV, and then different parts of rice plants were sampled for qRT-PCR analyses. The results showed that after RSV inoculation at the bottom of the ZD88 stem, the expression of *STV11* was first induced at the inoculated position and was relatively stable at the non-inoculated position (Figure 4C), suggesting that the expression of *STV11* responds to RSV infection only at the invasion site rather than at the whole plant. The transcription level of *STV11* in the rice stem bottom 7 dpi was higher than at other time points, and then decreased sharply (Figure 4C). At this time, virus accumulation in the rice stem bottom also decreased significantly 14 dpi (Figure 4A,C). Over time, RSV moved to the non-inoculated sites, and the accumulation of virus increased significantly with time; however, the expression of *STV11* showed little difference and still showed a downward trend (Figure 4A,C). These results indicate that *STV11* directly inhibited the accumulation of the virus at the invasion position.

In summary, *STV11* in ZD88 was continuously highly expressed in the RSV-infected position, and resistance to RSV was achieved by inhibiting the accumulation of virus in the rice-infected position.

### 2.4. Inoculating Rice Black-Streaked Dwarf Virus (RBSDV) Using the Determined-Part Inoculation Approach

**Figure 5:**
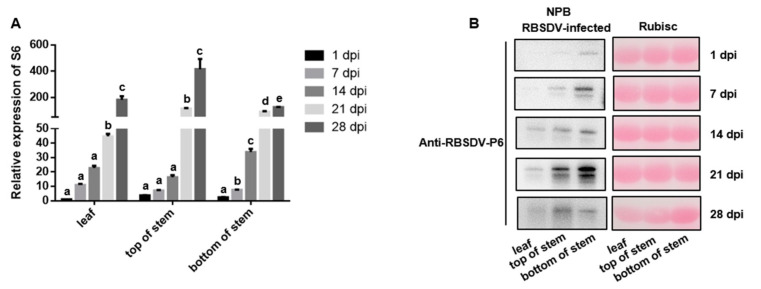
*Rice black-streaked dwarf virus* (RBSDV)-P6 accumulation in different parts of NPB. (**A**) Relative expression of RBSDV-P6 in different parts of NPB plants based on qRT-PCR analyses in which the average expression level of RBSDV-S6 in the leaves was set as 1 to estimate relative levels of gene expression in other parts of plants. (**B**) The expression level of RBSDV-P6 in different parts of NPB plants by Western blot. All data are shown as mean values ± SD error bars; different letters on bar graphs indicate significant differences by Duncan’s multiple range tests; *p* < 0.01.

To evaluate whether our method can also be used to inoculate rice plants with other rice viruses, RBSDV, another rice virus transmitted by SBPH, was inoculated on the bottom stem of NPB using the determined-part inoculation approach, and the transcription and accumulation levels of RBSDV-P6 were detected during the infection process. The results showed that from the initial day of inoculation, the accumulation of RBSDV-P6 in the NPB plant bottom stem increased significantly every 7 days from the first day after inoculation and reached a peak at 28 days after inoculation (Figure 5A). RBSDV-P6 at NPB-inoculated sites showed a continuous growth trend, indicating that this approach can also show the accumulation of RBSDV-P6 at the invasion site.

The accumulation of RBSDV-P6 protein was detected 1 day after inoculation with RBSDV at the bottom of the NPB stem, and at 14 and 21 days after inoculation RBSDV-P6 was detected at the top of the NPB stem and leaf (Figure 5B). The results demonstrated that after inoculation at the bottom of the stem, RBSDV moved from the infection position to the leaf, which indicated that RBSDV had moved in the plant, and this approach can show the transport of other rice viruses from the invasion site to other parts of the plant.

## 3. Discussion

Local and systematic viral infection processes are usually studied by inoculating the virus through an infectious clone or through mechanical friction on a single leaf a plant, and then viral accumulation is detected in inoculated and non-inoculated leaves [28]. However, in most cases, this method is only suitable for dicotyledons because it may be difficult to control the inoculation sites precisely on monocotyledons, such as rice. Most rice viruses are insect-borne [22], and the current inoculation method for rice viruses is a whole-plant inoculation method based on viruliferous insect feeding [26]. Although this method has been widely used in rice resistance studies, the initial inoculation site of rice viruses cannot be accurately controlled by this method, which limits further research on the infection processes of rice viruses. To solve this problem, a determined-part inoculation method is established in this study. This method can be used to simultaneously observe three key steps of viral infection process: virus invasion, viral accumulation at invasion sites, and spread in other plant parts. First, RSV was used as a model rice virus to evaluate this method. The feeding of viruliferous SBPH was restricted to the bottom of the stem of rice plants, and RSV-encoded CP could only be detected at the inoculation site at 1 dpi (Figure 2B). Over time, RSV accumulated at the inoculation site and spread to other parts of the rice plants, and RSV-CP and viral RNA were detected in the top of the stem and leaf at 7 and 14 dpi (Figure 2A,B), respectively. These results demonstrated that the initial invasion of RSV could be controlled at the inoculation site, and local accumulation and systemic spread could also be monitored.

In a previous study, the RSV resistance gene *STV11* was reported to sulfonate SA into SSA to confer durable RSV resistance and inhibit RSV replication in the protoplast [5]. However, the resistance of *STV11* to RSV was analyzed using the traditional whole-plant inoculation method, and whether *STV11* also inhibit the accumulation of RSV in rice plants and the distribution of the virus in different parts of rice plants are still unclear. In our study, by comparing the viral accumulation in NPB and ZD88 (carrying the *STV11* gene) using the determined-part inoculation method, we found that the local accumulation of RSV was inhibited in ZD88 from the initial invasion stage to the late stage, while the tissue specificity may not be affected. In addition, the systemic spread of RSV may not be affected, and the virus can still be detected in the leaves of plants. Our results confirm those of Wang et al. at the rice plant level and support their conclusions. This finding confirms that *STV11* confers resistance on RSV by inhibiting viral accumulation at the plant level, whereas viral systemic movement might not be affected by *STV11*, which is in accordance with a previous report [5]. Compared with the whole-plant inoculation method, our method can be used to compare viral invasion and systemic spread between susceptible and resistant cultivars, which may provide useful clues for the study of resistance mechanisms.

Wang et al. (2014) also found that the expression of *STV11* was induced in response to RSV infection. At present, there are no reports on the interaction between *STV11* and RSV-encoded proteins; therefore, when and where *STV11* is upregulated after RSV infects the plant is largely unknown. We found that in ZD88, the expression level of *STV11* was highly induced at the inoculation position at the initial invasion stage of the virus (1 dpi) and reached a peak at 7 dpi (Figure 4C); whereas the expression of *STV11* was only moderately upregulated in leaves from 14 to 21 dpi (Figure 4C), which agreed with the process of RSV infection (Figure 4A,B). These results indicate that although *STV11* was reported to be induced upon RSV infection, it was only highly induced at the RSV initial invasion site (the bottom of the stem in our study) to inhibit virus accumulation. Our results deepen our understanding of rice resistance gene expression patterns during viral infections.

It is challenging to increase both disease resistance and yield potential in crop breeding [29]. Enhancing the defense against diseases usually impairs the growth and development of the plant, posing a trade-off between growth and defense [30]. Interestingly, the precise regulation strategy of *STV11* mentioned above can serve to balance the host’s resistance to pathogens and their own growth and development needs, which is a manifestation of co-evolution between the plant virus and host. Therefore, the specific expression of *STV11* in rice in response to viral infection can maintain the relative balance in grain yield. This may be the reason why disease-resistance genes can be used in production for a long time [5,11].

Most of the plant viruses are insect-borne viruses [31], and in our study we prove that use of the determined-part inoculation method can also monitor the infection process of RBSDV, another representative rice virus also transmitted by SBPH. This suggests that our method may be applied universally for the inoculation of other insect-borne rice viruses. We also compared the viral infection processes between RSV and RBSDV in NPB. Interestingly, we found that the spread rate of RBSDV was much lower than that of RSV. In non-inoculated sites, the transcription level of RSV-CP at the top of the stem and leaf was significantly higher than that at 1 and 7 days after inoculation and reached a peak at 21 days after inoculation (Figure 2A). The transcription level of RBSDV-P6 at the top of the stem and leaf showed no significant change from 1 to 14 days after inoculation but increased significantly at 21 and 28 days after inoculation (Figure 5A). These results are in accordance with the observation in field trials that it takes longer for RBSDV to show symptoms in rice after inoculation compared with RSV.

For diseases caused by fungi, bacteria, nematodes, and some viruses, the inoculation sites on plants can be controlled, and there are many relevant reports about the parts of the host plants susceptible to these pathogens. Inoculating pathogens at a susceptible position is beneficial for improving the efficiency of inoculation and facilitating the study of the disease development process, as well as for proposing corresponding prevention and control measures, such as application of pesticides to susceptible parts [32]. However, there are few reports on the parts of rice plants susceptible to rice viruses, and the most critical constraint is the limitation of inoculation methods. The determined-part inoculation method established in this study is capable of controlling the initial invasion site of rice viruses, providing a useful tool to identify the susceptible parts of rice plants in future investigations.

## 4. Materials and Methods

### 4.1. Plant Materials and Virus-Infected Plants

The wild-type rice (*Oryza sativa* L. ssp. japonica) cultivars Nipponbare (NPB) and Wuyujing No. 3 were used in this study as highly susceptible to RSV. A highly resistant cultivar to RSV, *O. sativa* L. japonica Zhendao88, was also used in this study. RBSDV-infected cultivar Huaidao No. 5 was planted in the Jiangsu Academy of Agricultural Sciences (Nanjing, China). All plants were grown in a glasshouse at 28–30 °C with a 14 h light/10 h dark cycle under artificial light.

### 4.2. Feeding and Dot-Enzyme Linked Immunosorbent Assay (ELISA) of Insect Vector SBPH

The vector of RSV and RBSDV was SBPH. The second-instar virus-free SBPHs were fed the RSV-infected cultivar Huaidao No. 5, planted by the Jiangsu Academy of Agricultural Sciences. After 7 days of feeding, the SBPHs carrying RSV were tested using a Dot-ELISA assay to test the viruliferous SBPH rate. The second-instar SBPH nymphs were allowed to feed on RBSDV-infected Huaidao No. 5 rice plants for 7 days and then on the WT rice plants for 8 days. RBSDV viruliferous SBPHs were tested using a Dot-ELISA assay to test the viruliferous SBPH rate. In the Dot-ELISA assay, the monoclonal antibodies RSV-CP and RBSDV-P6 for RSV and RBSDV were provided by Professor Jianxiang Wu of Zhejiang University, and the titer of the monoclonal antibody was 1:5000. Viruliferous or virus-free SBPHs were reared on healthy rice seedlings (Wuyujing No. 3) grown in a 1 L beaker at 25 °C.

### 4.3. RSV Inoculation and Sampling Process

RSV was inoculated by two methods: one involved the inoculation of the whole plant, the process described previously [3,26], and the other method involved determined-part inoculation as mentioned in Figure 1. Briefly, rice seedlings at the 1.5- to 2.0-leaf stages were inoculated with SBPHs carrying RSV, and three viruliferous SBPHs were allowed to feed on each whole plant or the bottom (2 cm from soil surface) of the plant stem. After 3 days, all insects and tubes containing cotton were completely removed. The inoculated plants were grown in a greenhouse to induce symptoms. For each independent experiment, at least three biological replicates of each treatment and at least 35 seedlings were used for each replicate. Infected plants with RSV exhibited characteristics such as yellow-green leaf color and new leaf curl at 21 dpi. qRT-PCR with virus-specific primers RSV-CP-QF and RSV-CP-QR (Supplemental Table S1) was used to confirm the disease incidence in the inoculated plants. At 1, 7, 14, 21 and 28 days post-inoculation, samples were taken from the leaf, top of the stem (non-inoculated site), and bottom of the stem (inoculation site) of the plants, and the whole plant was sampled as a control for the second result. At each time point, at least three biological replicates of each sample and each replicate contained ten plants.

### 4.4. RNA Extraction and Quantitative Real-Time Polymerase Chain Reaction (qRT-PCR) Analysis

Total RNA of different rice parts samples were extracted using TRIzol reagent (Invitrogen) according to the manufacturer’s instructions. The concentration of each RNA sample was measured and adjusted the concentration to 500 ng/ul uniformly. The Takara PrimeScriptTM RT reagent Kit with gDNA Eraser was used to reverse transcribe 2 micrograms of each RNA sample. Quantitative real time qRT-PCR analysis was performed using the SYBR Premix mix as instructed (Takara, Japan). The rice 18s-rRNA was used as an internal control and normalization to calculate fold changes in gene expression during qRT-PCR. Relative expression levels of RSV-CP and *STV11* were analyzed by the 2^−ΔΔC(t)^ method [33]. At least three biological replicates with four technical replicates were used for each treatment, and each biological sample contained 10 mixed plants. Primers used in this experiment are listed in Table S1.

### 4.5. Protein Extraction and Western Blot Analysis

The different parts of the collected plants were ground individually in liquid nitrogen, and then mixed in a radio immunoprecipitation assay (RIPA Lysis Buffer, Beyotime P0013-B) supplemented with protease inhibitors (PMSF, final concentration is 1 mM). After centrifugation at 1200× *g* at 4 °C for 15 min, the supernatant was collected from each sample, and SDS-PAGE protein loading buffer (6×) was added to make the final concentration at 1×, and then the mixture liquid was boiled for 10 min. Proteins were separated on 10% sodium dodecyl sulfate polyacrylamide gel electrophoresis gels. Finally, they were transferred to PVDF membranes (activated with methanol for 15 s before transfer). An antibody against RSV-CP was used to detect the viral coat protein of samples.

### 4.6. Statistical Analysis

Each experiment undertook at least three biological replicates, and each biological sample had at least three technical replicates. The data of the three biological samples are expressed as the mean ± SD. The significant difference between the control and treatment groups in each experiment was determined by Student’s *t*-test. Statistical significance was set at *p* ≤ 0.05. All analyses were performed using IBM SPSS Statistics 19 and GraphPad Prism 6.

## 5. Conclusions

This study is the first report on a determined-part inoculation method, which is capable of controlling the initial infection of viruses in rice. By using this method, we monitored the local accumulation and systemic spread of RSV in NPB and ZD88 rice plants and confirmed that RSV replication was inhibited in ZD88 at the plant level. Moreover, we found that the expression of resistance gene *STV11* was significantly induced during the early invasion stage of RSV. The new method described in this study is also suitable for inoculation of other rice viruses, such as RBSDV, and it can be used to compare the infection processes of different rice viruses. The method and results presented in this study not only facilitate our discovery of the basic principles of plant biology but are also important for the study of host viral resistance mechanisms and to prevent and treat viral diseases.

## Data Availability

Not applicable.

## Supplementary Materials

The following supporting information can be downloaded at: https://www.mdpi.com/article/10.3390/pathogens11020144/s1. Supplemental Table S1. Primer information. Supplemental Figure S1. The initial invasion site of the virus after the whole plant is inoculated with RSV. Supplemental Figure S2. Comparison of virus accumulation in different parts of RSV invaded NPB and Zhendao 88.
