# Supplementary material for: Analyses on the Infection Process of Rice Virus and the Spatiotemporal Expression Pattern of Host Defense Genes Based on a Determined-Part Inoculation Approach
Source: Pathogens. 2022 Jan 24;11(2):144. doi: 10.3390/pathogens11020144 (PMC8880328; doi:10.3390/pathogens11020144)
Supplement: Supplementary file 1 [file pathogens-11-00144-s001.zip › pathogens-1521465-supplementary.pdf]

Supplemental Figure S1

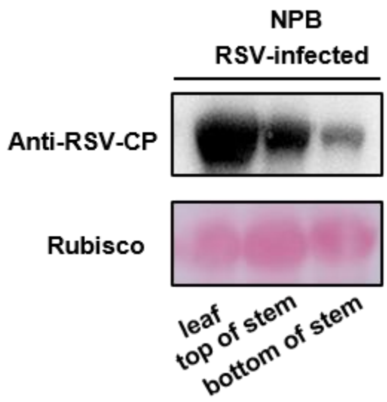

Figure S1. RSV accumulation in different parts of the plant, 1 day post-inoculation of RSV in the whole plants. The expression level of RSV-CP in different parts of NPB plants by Western blot.

Supplemental Figure S2

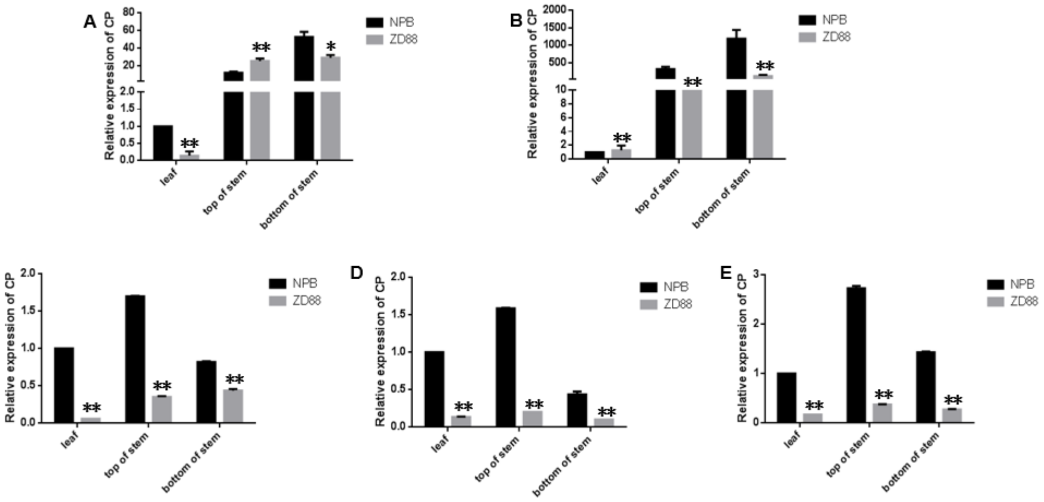

Figure S2. Comparison of RSV-CP accumulation in different parts of RSV-infected NPB and Zhendao 88. (**A**) Comparison of RSV-CP accumulation in different parts at 1 dpi. (**B**) Comparison of RSV-CP accumulation in different parts at 7 dpi. (**C**) Comparison of RSV-CP accumulation in different parts at 14 dpi. (**D**) Comparison of RSV-CP accumulation in different parts at 21 dpi. (**E**) Comparison of RSV-CP accumulation in different parts at 28 dpi. All data are shown as mean values ± SD error bars; $p \leq 0.05$ was considered statistically significant, and $p \leq 0.05$ or 0.01 were denoted with * or ** respectively.
